# Zinc-Guided 3D Graphene for Thermally Chargeable Supercapacitors to Harvest Low-Grade Heat

**DOI:** 10.3390/molecules27041239

**Published:** 2022-02-12

**Authors:** Qi Wang, Pengyuan Liu, Fanyu Zhou, Lei Gao, Dandan Sun, Yuhang Meng, Xuebin Wang

**Affiliations:** National Laboratory of Solid State Microstructures (NLSSM), Collaborative Innovation Center of Advanced Microstructures, Jiangsu Key Laboratory of Artificial Functional Materials, College of Engineering and Applied Sciences, Nanjing University, Nanjing 210093, China; qi_wang@smail.nju.edu.cn (Q.W.); Liupengyuan132@163.com (P.L.); 15655104861@163.com (F.Z.); dg21340032@smail.nju.edu.cn (L.G.); sdandan988@163.com (D.S.); myuh777@163.com (Y.M.)

**Keywords:** 3D graphene, supercapacitor, thermoelectric conversion, low-grade heat energy

## Abstract

Low-grade heat energy recycling is the key technology of waste-heat utilization, which needs to be improved. Here, we use a zinc-assisted solid-state pyrolysis route to prepare zinc-guided 3D graphene (ZnG), a 3D porous graphene with the interconnected structure. The obtained ZnG, with a high specific surface area of 1817 m^2^·g^−1^ and abundant micropores and mesopores, gives a specific capacitance of 139 F·g^−1^ in a neutral electrolyte when used as electrode material for supercapacitors. At a high current density of 8 A·g^−1^, the capacitance retention is 93% after 10,000 cycles. When ZnG is used for thermally chargeable supercapacitors, the thermoelectric conversion of the low-grade heat energy is successfully realized. This work thus provides a demonstration for low-grade heat energy conversion.

## 1. Introduction

In response to the climate crisis, major economies have announced their own plans. In its actual implementation, efficient use of existing energy is very important [1,2,3,4,5,6]. At present, heat is the principal way of energy supply; however, more than 60% of it is dissipated [7]. According to the heat source of different temperatures, waste-heat power generation is mainly divided into the steam Rankine (340–370 °C), organic Rankine (150–300 °C), Kalina (100–450 °C), and supercritical CO_2_ power cycles (225–650 °C) [8]. However, the utilization of low-grade heat energy (<100 °C) is still in the laboratorial research stage [9]. According to different principles and devices, there are two main types of low-grade thermal energy utilization, i.e., thermocells and thermally chargeable supercapacitors (TCSs) [10,11,12]. Compared with thermocells, the advantages of TCSs, such as simple equipment, longer cycle life, and wide temperature range, reveal broad application prospects [13,14].

Electrode material is one of the most important components of supercapacitors and plays an important role in TCSs [15,16,17]. Carbon materials have been widely studied due to their various forms, wide sources, good conductivity, and excellent stability [18,19,20]. Owing to the principle of the energy storage mechanism based on the electric double layer, carbon materials show a positive correlation between the specific capacitance and the specific surface area when applied to supercapacitors [21,22,23]. If the supercapacitor is thermally charged, it is manifested that the expansion of the electric double layer leads to an increase in the voltages of devices, thereby realizing thermoelectric conversion [24,25]. Qiao et al. found that the specific surface area of the electrode has an important influence on the generation of thermoelectric potential [26]. Bonetti et al. found that although the Seebeck coefficient of Pt is larger, the energy conversion efficiency of the porous carbon electrode is larger than that of Pt because the specific capacitance of the porous carbon electrode is much larger [27]. Therefore, a high specific surface area and a high specific capacitance will have a beneficial impact on the performance of TCSs. However, it is still difficult to prepare carbon material with a high specific surface area for TCSs.

Recently, our group has developed a unique method for preparing zinc-guided 3D graphene (ZnG) via a zinc-assisted solid-state pyrolysis (ZASP) route [28]. In this method, zinc with a low boiling point (907 °C) is used as the tiering agent, and glucose is used as the carbon source. In the preparation stage, zinc can penetrate and tier the char. The obtained ZnG has a structure of pure graphene membranes, showing a large specific surface area. ZnG is further applied to the TCSs, which show a high specific capacitance and long cycle life. The Seebeck coefficient of ZnG-based TCSs is 0.66 mV·K^−1^, higher than that of the commercial activated carbon (0.44 mV·K^−1^). The thermoelectric conversion of the low-grade heat energy is thus realized.

## 2. Results

The 3D graphene, ZnG, is prepared via the ZASP route. Cheap and readily available glucose was used as the carbon source, and the low-boiling zinc powder was used as the tiering agent. With the increase in temperature, glucose was transformed into the intermediate, char, which the zinc gradually penetrated. Driven by surface tension, the char was tiered, and the char lamella became the graphene membrane at 1000 °C. Finally, ZnG consisting of pure graphene membranes was prepared.

**Figure 1:**
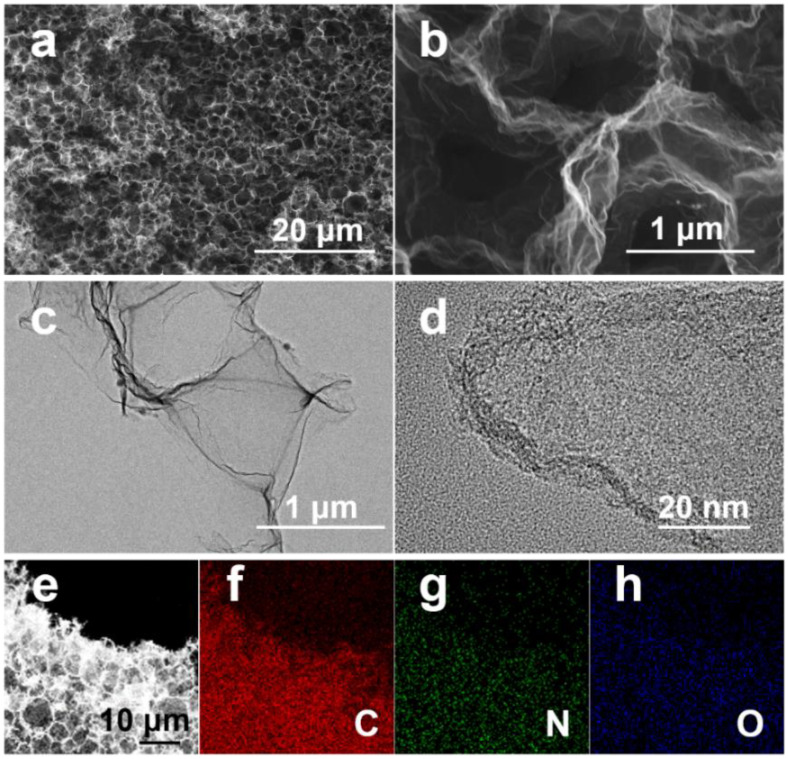
Morphology of ZnG. SEM images (**a**,**b**,**e**), TEM images (**c**,**d**), and elemental mapping of C (**f**), N (**g**), O (**h**).

The morphology of the prepared ZnG is shown in Figure 1. ZnG exhibits an interconnected porous structure (Figure 1a). ZnG is composed of curled thin graphene membranes (Figure 1b). The thin-layer graphene structure of ZnG is further characterized by TEM (Figure 1c). The high-resolution TEM, as shown in Figure 1d, confirms that the thickness of the curled graphite layer is 2.5–5.8 nm. The 3D porous network structure of graphene membranes can not only improve the accessibility of the ZnG surface for electrolyte but also facilitate the migration rate of ions within pores of electrode materials. It can also facilitate the rapid transport of electrons. The above advantages lay the foundation for excellent supercapacitor performance. Lastly, the elemental composition of ZnG is detected by EDS. Figure 1e–h clearly shows the presence of C, N, and O elements. The zinc element is not detected in ZnG, which indicates that the zinc was volatilized after heat treatment at 1000 °C.

**Figure 2:**
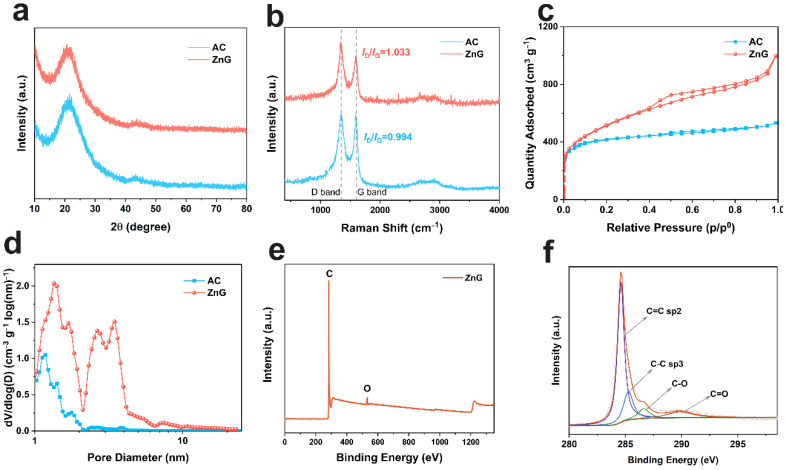
Spectroscopic characterization of ZnG and AC. XRD patterns (**a**); Raman spectra (**b**); nitrogen adsorption–desorption isotherms (**c**); corresponding pore size distributions (**d**); XPS survey spectrum (**e**); and high-resolution XPS spectrum of C 1s (**f**).

In order to illustrate the characteristics of ZnG, it was compared with the control specimen, commercial activated carbon. Figure 2a shows the XRD patterns of ZnG and AC. Two broad peaks appear around 21° and 44°, representing (002) and (100) crystal planes, respectively [29,30]. The defects are further characterized by Raman spectra, and two obvious peaks appear in Figure 2b [31]. The D band representing the disordered carbon structure and the G band representing the *E*_2g_ vibration mode appear at 1340 and 1590 cm^−1^, respectively. The intensity ratio of *I*_D_/*I*_G_ indicates the degree of defects [32,33]. It is 1.033 for ZnG, higher than that of AC (0.994). The band near 2670 cm^−1^ is the 2D band, i.e., the second-order Raman peak of the resonant scattering from the zone boundary. The low intensity of the 2D band of ZnG is usually attributed to the multilayer graphene stack [34]. The specific surface area and the pore structure of ZnG and AC are compared via a nitrogen adsorption and desorption test (Figure 2c). The specific surface area of ZnG is as high as 1817 m^2^·g^−1^, much larger than 1578 m^2^·g^−1^ of AC. Detailed pore size analysis shows that ZnG and AC have many micropores (Figure 2d), and ZnG also has many mesopores thanks to the delamination of the zinc. Therefore, ZnG shows the larger surface area and the greater mesoporous volume than AC. The presence of mesopores can provide more channels for the migration of ions in supercapacitors [35]. The elemental composition is further characterized by X-ray photoelectron spectroscopy (XPS). Only C and O elements are observed, and their contents are 95.3 and 4.7 at%, respectively (Figure 2e). This indicates that zinc is completely removed. The C 1s high-resolution XPS spectrum is deconvoluted as C=C (sp^2^, 284.6 eV), C−C (sp^3^, 285.3 eV), C−O (286.7 eV), and C=O (289.8 eV), and their contents are 63.1, 18.4, 7.8, and 10.7 at%, respectively (Figure 2f) [36].

**Figure 3:**
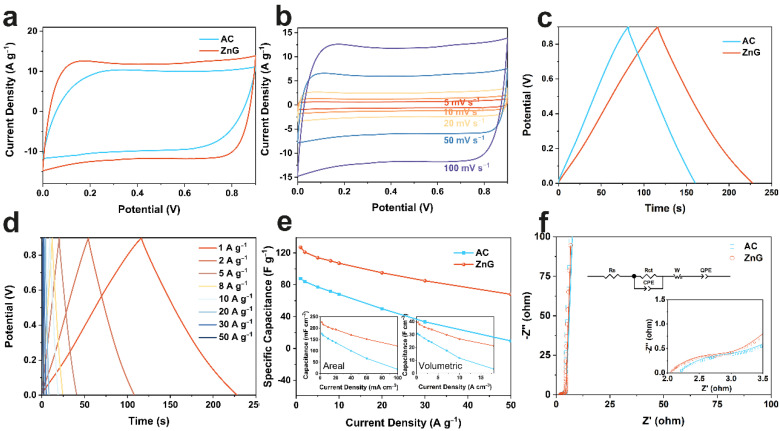
Electrochemical performance of ZnG and AC in three-electrode system. CV curves at 100 mV·s^−1^ (**a**); CV curves of ZnG at different scan rates (**b**); GCD profiles at 1 A·g^−1^ (**c**); GCD profiles of ZnG at different current densities (**d**); specific capacitance at different current densities, which insets are areal and volumetric capacitance (**e**); and Nyquist plots with the equivalent circuit (**f**).

The interconnected thin-layer graphene structure, the large specific surface area, and the microporous and mesoporous volume encourage ZnG to be a candidate electrode material for supercapacitors. At room temperature, the electrochemical performances of ZnG are evaluated in the three-electrode system. The electrolyte is 1 M KNO_3_, the counter electrode is platinum, and the reference electrode is the calomel electrode. First, the CV curves of ZnG and AC at a scan rate of 100 mV·s^−1^ are compared, where ZnG shows an approximately rectangular curve (Figure 3a). It indicates that the energy storage is dominated by the electric double-layer mechanism [37,38]. At the same current density, ZnG shows a larger encircled area, revealing a larger specific capacitance. Then, the CV curves of ZnG at different scanning rates are tested (Figure 3b). While the encircled area increases gradually, it still maintains a very similar rectangular shape, showing the good rate of performance of the ZnG electrode. The electrochemical properties of ZnG and AC are further analyzed by GCD test at 1 A·g^−1^ (Figure 3c). They also show the ideal electric double-layer behavior, where the GCD curve presents an approximate triangle [39]. The specific capacitance of ZnG is 124 F·g^−1^, larger than that of AC (89 F·g^−1^). By testing the GCD curves of ZnG at different current densities (Figure 3d), it is found that ZnG shows a triangular shape even at up to 50 A·g^−1^, revealing excellent high-rate capability. As shown in Figure 3e, the specific capacitances of ZnG are 127, 121, 114, 110, 107, 95, 85, and 68 F·g^−1^ at 1, 2, 5, 8, 10, 20, 30 and 50 A·g^−1^, respectively. The specific capacitance of ZnG is higher than that of AC at the same current density. Even at a current density of 50 A·g^−1^, the capacitance retention is still 54%, much higher than 11% of AC. Meantime, the areal and volumetric capacitances of ZnG electrodes are higher than AC ones at the same current density. Finally, the kinetics of electrode materials are characterized by the electrochemical impedance spectrum (EIS) (Figure 3f). The EIS curves are fitted with an equivalent circuit, which consists of ohmic resistance (*R*_s_), charge transfer resistance (*R*_ct_)/Faradaic leakage resistance (*R*_F_), Warburg diffusion resistance (w), double-layer capacitance (CPE), and the Faradaic capacitance (QPE) [40,41]. The intersection point at the transverse axis represents *R*_s_, the semicircular arc at the high-frequency region represents *R*_ct_/*R*_F_, and the straight line at low frequency relates to the diffusion of ions [42]. *R*_s_ and *R*_ct_/*R*_F_ are 2.04 and 0.74 Ω for ZnG and 2.17 and 0.91 Ω for AC, respectively. The ZnG electrode shows smaller *R*_s_ and stronger ion diffusion ability, because the 3D interconnected structure improves the electron and ion transport ability, and the mesopores further improve ion diffusion ability.

**Figure 4:**
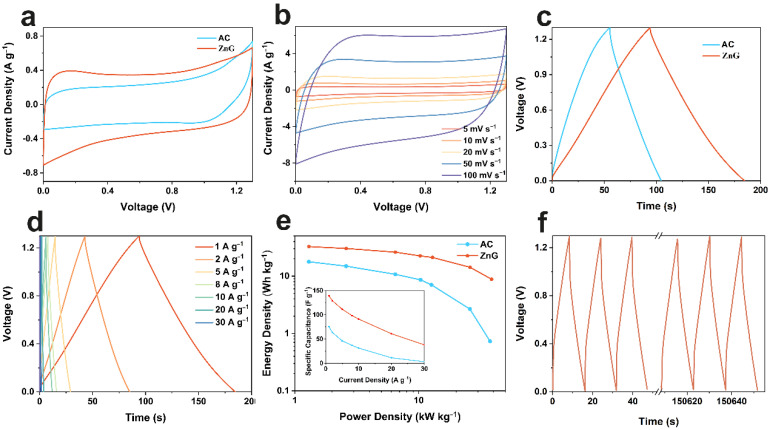
Electrochemical performance of ZnG and AC in symmetric supercapacitor. CV curves at 5 mV·s^−1^ (**a**); CV curves of ZnG symmetric supercapacitor at different scan rates (**b**); GCD profiles at 1 A·g^−1^ (**c**); GCD profiles of ZnG symmetric supercapacitor at different current densities (**d**); Ragone plots and specific capacitance at different current densities (**e**); and the first and the final three cycles of cycling tests (**f**).

Considering the excellent performance of ZnG in the neutral electrolyte, we assembled the symmetric supercapacitor. The shape of the approximate rectangle is also seen in the CV test of the two-electrode system (Figure 4a), which again confirms the electric double-layer energy storage mechanism. Through the comparison of CV curves at different scanning rates (Figure 4b), the rectangular shape is maintained at a high scanning rate, indicating its excellent rate performance. Figure 4c shows the galvanostatic charge and discharge (GCD) curves of ZnG and AC at 1 A·g^−1^, with specific capacitances of 139 and 76 F·g^−1^, respectively. Then, the GCD curves of the ZnG symmetric supercapacitor at different current densities are tested. The specific capacitances of the ZnG symmetric supercapacitor are 139, 129, 112, 98, 91, 61, and 38 F·g^−1^ at 1, 2, 5, 8, 10, 20, and 30 A·g^−1^, respectively. As shown in Figure 4e, the ZnG symmetric supercapacitor shows a higher energy density than that of AC. When the power density is 39 kW·kg^−1^, the energy density is still 9 Wh·kg^−1^. Finally, the stability of the symmetric supercapacitor is tested (Figure 4f). At a current density of 8 A·g^−1^, the ZnG symmetric supercapacitor remains 90 F·g^−1^ after 10,000 cycles, higher than 36 F·g^−1^ of AC.

**Figure 5:**
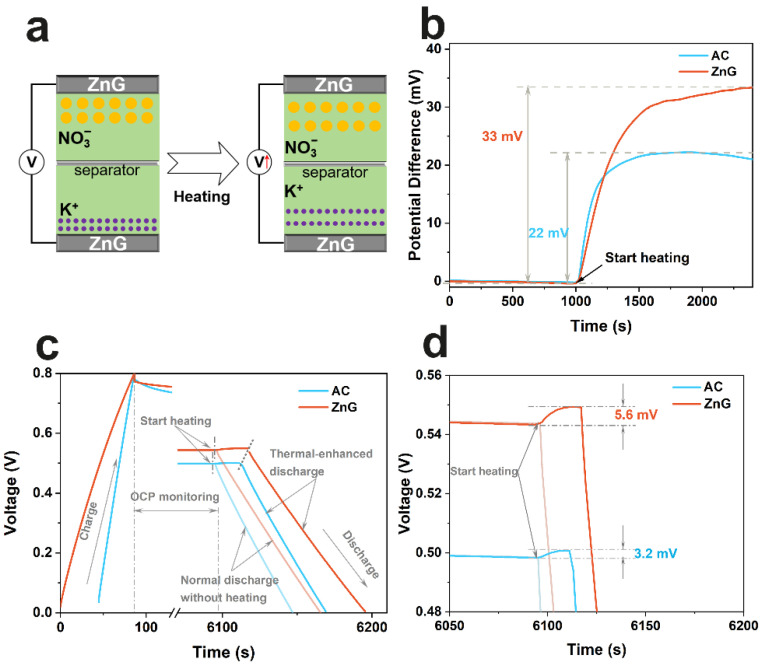
Comparison of thermal conversion ability between ZnG and AC. Schematic illustration of thermal charging mechanism, where the thermally induced electric double layer expansion increases the voltage (**a**); Seebeck coefficient tested at H-type electrochemical cell (**b**); charging–stabilizing–heating–discharging tests at 0.5 A·g^−1^ (**c**); the amplification of the heating–discharging part (**d**).

The performance of ZnG in the application of thermal charging supercapacitors is further explored. As shown in Figure 5a, the electric double layer forms on the electrode surface after charging. Heating can expand the electric double layer and can thus increase the voltage between electrodes. The capacity of the hot-state supercapacitors should indeed decline, but the total discharge of electricity will increase, thus realizing thermoelectric conversion [24]. The Seebeck coefficients of ZnG or AC are tested in Figure 5b. The electrolyte, the counter electrode, and the reference electrode are 1M KNO_3_, the platinum sheet, and the calomel electrode, respectively. When the working electrode is heated, the ratio of the change value of the open circuit potential to the change value of the temperature is viewed as the Seebeck coefficient. The Seebeck coefficients of ZnG or AC are 0.66 or 0.44 mV·K^−1^, respectively. The larger Seebeck coefficient of ZnG may be attributed to the enhanced conductivity of the electrode material, owing to the 3D interconnected structure [17]. Subsequently, the charging–stabilizing–heating–discharging tests of the ZnG or AC symmetric supercapacitors are carried out in Figure 5c,d. The specific energy values generated by heating are 226 or 98 mJ·g^−1^ for ZnG or AC cases, respectively. The comparison of the thermal conversion ability of the ZnG material with other state-of-the-art materials is summarized in Table S1.The results reveal that ZnG shows stronger thermoelectric conversion ability than AC, which is mainly attributed to the large specific surface area and the 3D network structure of ZnG.

## 3. Materials and Methods

### 3.1. Preparation of ZnG

ZnG was prepared via zinc-assisted pyrolysis of glucose, as described in the literature [28]. Typically, zinc powders and glucose (m_Zn_:m_glucose_ = 9:1) were mixed in water, and the water was then dried to obtain zinc@glucose mixture. The mixture was molded under a pressure of 10 MPa to acquire a bulk zinc@glucose. The bulk was placed in the tubular furnace, and N_2_ was introduced. The temperature was raised to 1000 °C at 5 °C min^−1^, and the temperature was kept for 3 h to obtain ZnG. Activated carbon (AC) was supplied by Kuraray Co., Ltd (Okayama, Japan).

### 3.2. Electrochemical Tests

ZnG was used as an electrode material without conductive agents or binders. The preparation of the activated carbon electrode included the following steps: According to the mass ratio of AC, acetylene black, and polyvinylidene difluoride of 8:1:1 in N-methylpyrrolidone solvent, the slurry was coated on the surface of the graphite plate. The area mass loading of AC was 1 mg·cm^−2^. In− the three-electrode system, 1 M KNO_3_ was used as the electrolyte, the platinum sheet was used as the counter electrode, and the calomel electrode was used as the reference electrode. The potential window was 0–1 V. In the two-electrode system, two pieces of ZnG (1 mg) were placed on both sides of the separator to assemble a symmetric supercapacitor. Two AC-coated graphite plates were assembled similarly into a symmetric supercapacitor. All electrochemical tests were performed on CHI 660E ( Chenhua Instrument Co., Ltd., Shanghai, China), using open circuit potential (OCP) monitoring, galvanostatic charge and discharge (GCD), electrochemical impedance spectrum (EIS), and cyclic voltammetry (CV).

In the three-electrode system, the specific capacitance was calculated from GCD curves via

(1) $$C=\frac{i\times ∆t}{m_{1}\times ∆V}$$

where ∆*V*, *i*, and *m*_1_ are the potential window, the current, and the mass of active materials, respectively, and ∆*t* is the discharge time.

In symmetric supercapacitors, the specific capacitance *C*_s_, the energy *E*_s,_ and the power densities *P*_s_ were calculated from the following equations:

(2) $$C_{s}=\frac{4I}{m\left(dV/dt\right)}$$

(3) $$E_{s}=\frac{C_{s}V^{2}}{8}$$

(4) $$P_{s}=\frac{E_{s}}{t}$$

where *I* is the current in discharging, and *m* is total mass of both electrodes.

### 3.3. Characterization

For Seebeck coefficient measurements, the H-type electrochemical cell with hot and cold separation was used. ZnG electrodes were placed on the hot side, and the platinum counter electrodes and the reference electrodes were placed on the cold side. The hot side was heated by a constant-temperature water bath, and the cold side was cooled by a cold-water bath pumped continuously at 20 °C. A thermometer was used to measure the real-time temperature. The Seebeck coefficient was calculated by the ratio of the variation of OCP to the variation of the temperature in a range of 20–70 °C. In the charging–stabilizing–heating–discharging tests, after heating the symmetrical supercapacitor from 25 to 45 °C, the galvanostatic test was performed. To evaluate the thermoelectric conversion ability, the amount of the electric energy converted from the heat energy, *w*, is calculated via [7]

(5) $$w=\frac{1}{2}C_{H}\psi_{B}∆\psi$$

where *C*_H_ is the specific capacitance at room temperature, *ψ*_B_ is the voltage value before the heating, and Δ*ψ* is the voltage rise value caused by the heating.

The morphology and the element composition of the samples were analyzed by scanning electron microscope (SEM, Hitachi 8100, Tokyo, Japan) combined with energy dispersive X-ray spectrometer (EDS) (Thermo Fisher Scientific, Waltham, MA, USA), transmission electron microscope (TEM, FEI Tennai F20, Hillsboro, OR, USA) and X-ray photoelectron spectroscopy (XPS, ESCA LAB 250 Xi, Thermo Fisher Scientific, Waltham, MA, USA). The structures of the samples were determined by XRD (Bruker D8, Cu Kα radiation, Bruker, Mannheim, Germany) and Raman (Wintec alpha300A, WITec, Ulm, Germany). Specific surface area and pore size distribution were calculated using Brunauer–Emmett–Teller (BET) and density functional theory (DFT) models after nitrogen adsorption and desorption tests (autosorb iQ, Quantachrome, Boynton Beach, FL, USA).

## 4. Conclusions

In summary, 3D graphene with the interconnected structure, ZnG, is prepared via a zinc-assisted pyrolysis route. Because of the large specific surface area, the electrochemical performance of ZnG is significantly improved compared to the commercial activated carbon when applied to supercapacitors. The energy density of the ZnG-based symmetric supercapacitor in the neutral electrolyte is 9 Wh·kg^−1^ at a power density of 39 kW·kg^−1^. When applied to the thermally-chargeable supercapacitors, ZnG realizes the large thermoelectric conversion of low-grade heat energy (226 mJ·g^−1^). It thus contributes a demonstration to the field of thermoelectric conversion.

## Data Availability

The data presented in this study are available on request from the corresponding author.

## Supplementary Materials

The following are available online. Table S1: Comparison of thermal conversion ability of ZnG and other state-of-the-art materials [13,14,16,27,43,44,45,46,47,48,49,50,51,52,53].

## Sample Availability

Samples of the compounds are not available from the authors.
