# Supplementary material for: Zinc-Guided 3D Graphene for Thermally Chargeable Supercapacitors to Harvest Low-Grade Heat
Source: Molecules. 2022 Feb 12;27(4):1239. doi: 10.3390/molecules27041239 (PMC8880206; doi:10.3390/molecules27041239)
Supplement: Supplementary file 1 [file molecules-27-01239-s001.zip › molecules-1559882-supplementary.pdf]

molecules

MDPI

Supporting Information

Zinc-Guided 3D Graphene for Thermally Chargeable Supercapacitors to Harvest Low-Grade Heat

Qi Wang, Pengyuan Liu †, Fanyu Zhou †, Lei Gao †, Dandan Sun, Yuhang Meng and Xuebin Wang *

Table S1. Comparison of thermal conversion ability of ZnG and other state-of-the-art materials.

Electrodes	Electrolyte	Seebeck Coefficient (mV °C^{-1})	Temperature Difference (°C)	Specific Energy/Power Output	Reference
Rice husk char//rice husk char	20% NaCl	1.34	50	106.7 mJ g^{-1}	[1]
Carbon cloth//carbon cloth	0.1 M $K_3Fe(CN)_6/K_4Fe(CN)_6$ (PVA gel)	1.2	50	973.6 μJ cm^{-2}	[2]
Pt//304 stainless steel	0.4 M iodide/triiodide	0.26	30	—	[3]
NiNC//NiNC	1 M $NaC_2H_3O_2$ (FA)	2.5	45	1.8 mJ g^{-1}	[4]
Carbon cloth//carbon cloth	0.4 M $K_3Fe(CN)_6/K_4Fe(CN)_6$ (PAC gel)	0.75	20	95 mW m^{-2}	[5]
rSGO//rSGO	SGO (2M H_2SO_4)	4.53	14	894 μC cm^{-2}	[6]
Nanoporous carbon//Pt	1 M LiCl	0.68	40	—	[7]
Nanoporous carbon (Cabot BP2000)//Pt	0.1M LiCl	0.8	39	100 mJ g^{-1}	[8]
NanoTuneX Y-Carbon//Pt	2 M EMIMTFSI (AN)	−0.3	20	0.05 mJ m^{-2}	[9]
10 Farad supercapacitor (YEC Pl series)	$TEA\text{-}BF_4$ (acetonitrile)	0.6	65	185 mJ g^{-1}	[10]
Pt//Pt	Emim-Cl (PVA gel)	10	33	1.7 mJ g^{-1}	[11]
AC (YP-80F)//AC (YP-80F)	1 M KNO_3	1.21	52	—	[12]
P-G/CNT//P-G/CNT	Polystyrene sulfonic acid film	0.2	5	—	[13]
Porous carbon//porous carbon	1 M $NaC_2H_3O_2$ + Cu	5.3	50	4.2 W m^{-2}	[14]
Nanoporous graphene//nanoporous graphene	0.01 M KCl	4.54	35	1.38 mW·g^{-1}	[15]
ZnG//ZnG	1 M KNO_3	0.66	20	226 mJ g^{-1}	This work

References

1. Zhang, X.; Xu, D.; Xiong, Y. Effect of hydrothermal on thermoelectric properties of rice husk char. *Chem. Ind. Eng. Prog.* **2020**, *39*, 2632–2638.
2. Kundu, A.; Fisher, T.S. Harnessing the thermogalvanic effect of the ferro/ferricyanide redox couple in a thermally chargeable supercapacitor. *Electrochim. Acta* **2018**, *281*, 357–369.
3. Alzahrani, H. A.; Black, J.J.; Goonetilleke, D.; Panchompoo, J.; Aldous, L. Combining thermogalvanic corrosion and thermogalvanic redox couples for improved electrochemical waste heat harvesting. *Electrochem. Commun.* **2015**, *58*, 76–79.
4. Lim, H.; Shi, Y.; Qiao, Y. Thermally chargeable supercapacitor based on nickel-coated nanoporous carbon. *Int. J. Green Energy* **2018**, *15*, 53–56.
5. Buckingham, M.A.; Zhang, S.; Liu, Y.; Chen, J.; Marken, F.; Aldous, L. Thermogalvanic and Thermocapacitive Behavior of Superabsorbent Hydrogels for Combined Low-Temperature Thermal Energy Conversion and Harvesting. *ACS Appl. Energy Mater.* **2021**, *4*, 11204–11214.
6. Mageeth, A.M.A.; Park, S.; Jeong, M.; Kim, W.; Yu, C. Planar-type thermally chargeable supercapacitor without an effective heat sink and performance variations with layer thickness and operation conditions. *Appl. Energy* **2020**, *268*, 114975.
7. Lim, H.; Zhao, C.; Qiao, Y. Performance of thermally-chargeable supercapacitors in different solvents. *Phys. Chem. Chem. Phys.* **2014**, *16*, 12728–12730.
8. Lim, H.; Lu, W.; Chen, X.; Qiao, Y. Effects of ion concentration on thermally-chargeable double-layer supercapacitors. *Nanotechnology* **2013**, *24*, 465401.
9. Bonetti, M.; Nakamae, S.; Huang, B.T.; Salez, T.J.; Wiertel-Gasquet, C.; Roger, M. Thermoelectric energy recovery at ionic-liquid/electrode interface. *J. Chem. Phys.* **2015**, *142*, 244708.
10. Härtel, A.; Janssen, M.; Weingarth, D.; Presser, V.; van Roij, R. Heat-to-current conversion of low-grade heat from a thermocapacitive cycle by supercapacitors. *Energy Environ. Sci.* **2015**, *8*, 2396–2401.
11. Horike, S.; Wei, Q.; Kirihara, K.; Mukaida, M.; Sasaki, T.; Koshiba, Y.; Fukushima, T.; Ishida, K. Outstanding electrode-dependent Seebeck coefficients in ionic hydrogels for thermally chargeable supercapacitor near room temperature. *ACS Appl. Mater. Interfaces* **2020**, *12*, 43674–43683.
12. Chen, D.; Li, Z.; Jiang, J.; Wu, J.; Shu, N.; Zhang, X. Influence of electrolyte ions on rechargeable supercapacitor for high value-added conversion of low-grade waste heat. *J. Power Sources* **2020**, *465*, 228263.
13. Kim, S.L.; Lin, H.T.; Yu, C. Thermally Chargeable Solid-State Supercapacitor. *Adv. Energy Mater.* **2016**, *6*, 1600546.
14. Meng, T.; Xuan, Y. Enhancing Conversion Efficiency and Storage Capacity of a Thermally Self-Chargeable Supercapacitor. *Adv. Mater. Interfaces* **2020**, *7*, 2000934.
15. Yang, Z.; Dang, F.; Zhang, C.; Sun, S.; Zhao, W.; Li, X.; Liu, Y.; Chen, X. Harvesting Low-Grade Heat via Thermal-Induced Electric Double Layer Redistribution of Nanoporous Graphene Films. *Langmuir* **2019**, *35*, 7713–7719.
